# Extracellular calcium promotes bone formation from bone marrow mesenchymal stem cells by amplifying the effects of BMP-2 on SMAD signalling

**DOI:** 10.1371/journal.pone.0178158

**Published:** 2017-05-25

**Authors:** Rubén Aquino-Martínez, Natalia Artigas, Beatriz Gámez, José Luis Rosa, Francesc Ventura

**Affiliations:** Departament de Ciències Fisiològiques, Universitat de Barcelona, IDIBELL, L’Hospitalet de Llobregat, Spain; Medical University of South Carolina, UNITED STATES

## Abstract

Understanding the molecular events that regulate osteoblast differentiation is essential for the development of effective approaches to bone regeneration. In this study, we analysed the osteoinductive properties of extracellular calcium in bone marrow-derived mesenchymal stem cell (BM-MSC) differentiation. We cultured BM-MSCs in 3D gelatin scaffolds with Ca^2+^ and BMP-2 as osteoinductive agents. Early and late osteogenic gene expression and bone regeneration in a calvarial critical-size defect model demonstrate that extracellular Ca^2+^ enhances the effects of BMP-2 on *Osteocalcin*, *Runx2* and *Osterix* expression and promotes bone regeneration *in vivo*. Moreover, we analysed the molecular mechanisms involved and observed an antagonistic effect between Ca^2+^ and BMP-2 on SMAD1/5, ERK and S6K signalling after 24 hours. More importantly, a cooperative effect between Ca^2+^ and BMP-2 on the phosphorylation of SMAD1/5, S6, GSK3 and total levels of β-CATENIN was observed at a later differentiation time (10 days). Furthermore, Ca^2+^ alone favoured the phosphorylation of SMAD1, which correlates with the induction of *Bmp2* and *Bmp4* gene expression. These data suggest that Ca^2+^ and BMP-2 cooperate and promote an autocrine/paracrine osteogenic feed-forward loop. On the whole, these results demonstrate the usefulness of calcium-based bone grafts or the addition of exogenous Ca^2+^ in bone tissue engineering.

## Introduction

The combination of calcium-containing biomaterials and mesenchymal progenitors is regarded as a potential bone graft substitute in tissue engineering. In fact, bone is a heterogeneous biocomposite tissue that consists of an inorganic phase (essentially hydroxyapatite), an organic phase (mainly type I collagen) and cells [1] Physiological bone remodelling occurs when osteoblast precursors attach, differentiate and replace the bone previously resorbed by osteoclasts. Thus, osteoblast progenitors differentiate after exposure to multiple degradation products released by osteoclasts. The bone components released presented high extracellular calcium concentrations, collagen fragments and growth factors.

Functional type I collagen has a unique triple helical structure that uncoils during degradation to generate gelatin. This uncoiling exposes its RGD sequences, which promote integrin binding and cell attachment [2]. Unlike collagen, gelatin does not present antigenicity in physiological conditions [3]. These properties make gelatin an appropriate scaffold for use in 3D cell cultures [3]

Ca^2+^ levels differ significantly in different bone niches. Calcium from bone matrix is released in its ionic form into the remodelling microenvironment [4]. For instance, in the hemivacuole, studies have reported values in the range of 8–40 mM, in contrast to the non-resorbing surface of the osteoclast, where values were < 2 mM during resorption [5]. This local increment of Ca^2+^ concentration has a strong impact on the chemotaxis, proliferation and differentiation of osteoblasts [6]. Mechanistically, extracellular Ca^2+^ activates calcium-sensing receptors (CaSR). CaSRs are G protein-coupled receptors that are expressed in mesenchymal stem cells and osteoblasts [7], although their functional role in bone homeostasis remains unclear. Furthermore, voltage-gated Ca^2+^ channels increase intracellular Ca^2+^ levels in osteoblasts [8]. The maximum effects of extracellular Ca^2+^ on osteoprogenitor migration and differentiation are achieved at concentrations in the range of 2–10 mM [6]. However, although the osteoconductive or osteoinductive properties of Ca^2+^-containing scaffolds have been thoroughly exploited [9], little is known about their intracellular signal transduction in osteoblasts [10].

Osteogenic growth factors might be secreted by bone resident cells or released from bone matrix during bone remodelling. These factors that are released after resorption include several members of the TGFβ/BMP superfamily of osteogenic signals. TGFβ/BMP signals at the cell surface are transduced by the Smad family of transcription factors, which directly regulate gene expression and additional non-canonical pathways such as p38 MAP-kinase and phosphoinositide 3-kinase (PI3K)/AKT [11]. BMP target genes include a growing number of osteoblast-determining transcription factors such as *Dlx5*, *Runx2* and *Osterix* [12,13]. More importantly, the efficacy of BMPs in bone regeneration is well known in both animal models and clinical applications such as bone fracture healing [14], alveolar cleft defects [15], spinal fusion [16] and craniofacial bone defects [17,18]. As a result, BMP-2 and BMP-7 have been approved for medical use in specific osteoinductive applications.

Tissue engineered scaffolds for bone regeneration should lead to the attachment, propagation, and differentiation of the transplanted cells. Thus, there are three main components in the field of bone tissue engineering: a scaffold that provides structure and substrate for tissue growth, a source of progenitor cells and stimuli to direct growth and differentiation within the scaffold [9]. Despite early successes, many challenges are still faced. For instance, beside improvement of individual components, understanding the interactions between them is key for success [19]. We combined bone marrow-derived mesenchymal stem cells (BM-MSCs) and 3D gelatin scaffolds with Ca^2+^ and BMP-2 as osteoinductive agents. In an attempt to define the mechanisms of osteoinduction by Ca^2+^, we first assessed the potential effects of Ca^2+^ on osteoblast differentiation and bone formation and its cooperation with BMP-2. More importantly, we identified the molecular mechanisms involved in this cooperative osteogenic interaction. We propose that knowledge of these signalling events induced by Ca^2+^ could benefit the field of bone tissue engineering.

## Materials and methods

### Isolation and culture of bone marrow-derived mesenchymal stem cells (BM-MSCs)

BM-MSCs were isolated from the femurs of 6–8 week old BALB/c mice, as previously described [20,21]. Mice were euthanised and the femurs dissected. Next, the soft tissues were cleaned and the femurs kept in complete media (DMEM supplemented with 10% FBS, penicillin/streptomycin, 1 mM pyruvate and 2 mM glutamine). The femur ends were cut using a rongeur and the bone marrow was flushed and collected. Cell suspension was filtered with a 70 μm cell strainer (Falcon, USA), transferred to a 100 mm cell culture plate and incubated at 37°C. The media was changed after 24 hours and then every eight hours for two to three days to discard non-adherent cells. After five to seven days, when the adherent cells reached 75%-80% of confluence, the cells were washed three times with warmed PBS and trypsinised for three minutes at room temperature. The lifted cells were cultured and expanded for future experiments.

### 2D gelatin coating and 3D gelatin scaffold preparation

Twelve-well plate surfaces were coated with a thin layer of 0.1% gelatin/PBS solution. Plates were left for one hour inside a laminar flow hood to dry the treated surfaces and then stored until use. To prepare 3D scaffolds, a 1 mm^3^ gelatin sponge (Gelita, B. Braun) was used. Sponges were cut into 1 mm thick slices for *in vitro* assays, whereas 2 mm slices were used for the *in vivo* experiments. Scaffolds were soaked in complete media and incubated for 12–24 hours at 37°C. The BM-MSCs (2x 10^5^ cells in 20 μl of media per scaffold) were then seeded under sterile conditions into the gelatin scaffold, and everything was introduced into a microcentrifuge tube and incubated in a vertical position for four to six hours at 37°C. This seeding allowed efficient and equal attachment of the cells to the scaffold. To assess the effect of extracellular calcium on the osteoblast differentiation of BM-MSCs, CaSO_4_ or CaCl_2_ (Sigma-Aldrich) was used as source of Ca^2+^. BM-MSCs seeded on 3D gelatin scaffolds were cultured in the presence of different CaCl_2_ or CaSO_4_ concentrations in the culture media, which ranged from 3 mM to 10 mM, throughout the whole experimental period. Fresh media (100 μl) was added to the corresponding condition every three days.

### Gene expression analysis by RT-qPCR

RNA isolation was performed using Trisure (Bioline Reagents, UK) in accordance with the manufacturer’s protocol. The purified RNA was quantified using a spectrophotometer (Nanodrop). Two micrograms of RNA were retrotranscribed to cDNA using a High-Capacity cDNA Reverse Transcription Kit (Applied Biosystems) in accordance with the manufacturer’s protocol. The gene expression of *Alpl* (Mm00475834.m1), *Osteocalcin* (*Bglap2*) (Mm03413826.m1), *Runx2* (Mm03003491.m1), *Osterix(Sp7)* (Mm00504574.m1), *Bmp2* (Mm01340178.m1), *Bmp4* (Mm00432087.m1), *Fgf21* (Mm00840165.g1) and *Axin2* (Mm00443610.m1) was analysed using Taqman probes (Applied Biosystems) and normalised to *Gapdh* (Mm9999915.g1) expression by the 2ΔΔCt method.

### Western blot assay

BM-MSCs seeded in 3D gelatin scaffolds were cultured for 24 hours or 10 days. Cells were lysed with 75 μl of lysis buffer (PBS, 1% Triton X-100, 100 mM PMSF, 1 μg/ml pepstatin, 1 μg/ml leupeptin, 1 mM of sodium orthovanadate, 10 mM NaF and 10 mM β-glycerophosphate) for one hour at 4°C. Thirty micrograms of protein samples were subjected to SDS-PAGE and immunoblotting. Membranes were incubated with different antibodies: pGSK3α/β Ser9/21 (9331S), pSMAD1/5/8 Ser465/467 (9511S) and pS6 Ser235/236 (2211) and pp38 Thr180/Tyr182 (9211S) from Cell Signaling Technology, pErk1/2 (M5670) from Sigma, β-catenin (610154) from BD Transduction Laboratories and α-tubulin (T6199) from Sigma, all diluted to a ratio of 1:1000. Horseradish peroxidase-conjugated secondary antibodies were used, followed by incubation with EZ-ECL reagent (Biological Industries). A chemiluminescent image of the immunoblots was captured with a Fujifilm LAS 3000 device.

### Calvarial critical-size bone defects and in vivo bone regeneration

Procedures for animal experimentation were approved by the Animal Research Ethics Committee of the University of Barcelona and Generalitat de Catalunya. All methods in animal experiments were performed according to the approved institutional guidelines. A surgical procedure was performed in 10-week old male BALB/c mice. Animals were housed individually and fed *ad libitum*. Animals were anesthetised with isoflurane inhalation and an intraperitoneal injection of buprenorphine (0.05 mg/kg) was administered to provide intraoperative analgesia. To expose the parietal bones, a longitudinal midline incision was made and the tissues retracted. A circular critical-size bone defect with an outer diameter of 5 mm was carried out with a trephine bur on the left parietal. We cultured 4x 10^5^ BM-MSCs per scaffold in accordance with the protocol described above for 3D gelatin scaffold preparation. After cells in the 3D gelatin scaffolds had been exposed to the respective conditions for 48 hours, a 1% final concentration solution of low melting agarose at 36°C was added as a gelling agent. Scaffolds were implanted to fill the bone defects, depending on the respective experimental group. The incised tissues were sutured and the animals monitored daily during the recovery phase. Five weeks after surgery, the animals were euthanised and the calvariae dissected.

### Histological analysis

After skull fixation with 4% paraformaldehyde for 48 hours, the calvariae were decalcified with a Decalcifier II (Leica) for two days. They were then dehydrated, embedded in paraffin and cut into 6 μm sections to examine bone formation. Slides were stained using hematoxylin/eosin and Masson’s trichrome technique. Histological sections were evaluated using a light microscope (Nikon Eclipse E800). Quantification of the trichrome images was performed from the images at 4x magnification using ImageJ software.

### Statistical analysis

Data were was obtained from at least three independent experiments and presented as mean ± SEM. Data was analyzed for normality of the distribution by the Kolmogorov-Smirnov test. ANOVA (Bonferroni’s test) analysis was performed when more than two groups were compared. Differences were considered significant at *p* values of less than 0.05: ^*^p < .05, **p < .01, and ***p < .001.

## Results

### Extracellular calcium increases osteogenic gene expression in BM-MSCs cultured in 3D gelatin scaffolds

In order to evaluate the influence of the culture system on the osteoblast differentiation of BM-MSCs, we compared three different culture models: untreated plastic surface, 2D gelatin-coated surface and 3D gelatin scaffold. Cells seeded on 3D gelatin scaffolds showed greater upregulation of all osteogenic markers evaluated (*Alpl p*< 0.001; *Osteocalcin p*< 0.001 and *Osterix p*< 0.001) than monolayers on plastic surfaces or 2D gelatin coated plates (S1 Fig). This result suggests that 3D gelatin scaffolds promote higher osteoblast differentiation than plastic or 2D gelatin-coated surfaces.

**Fig 1:**
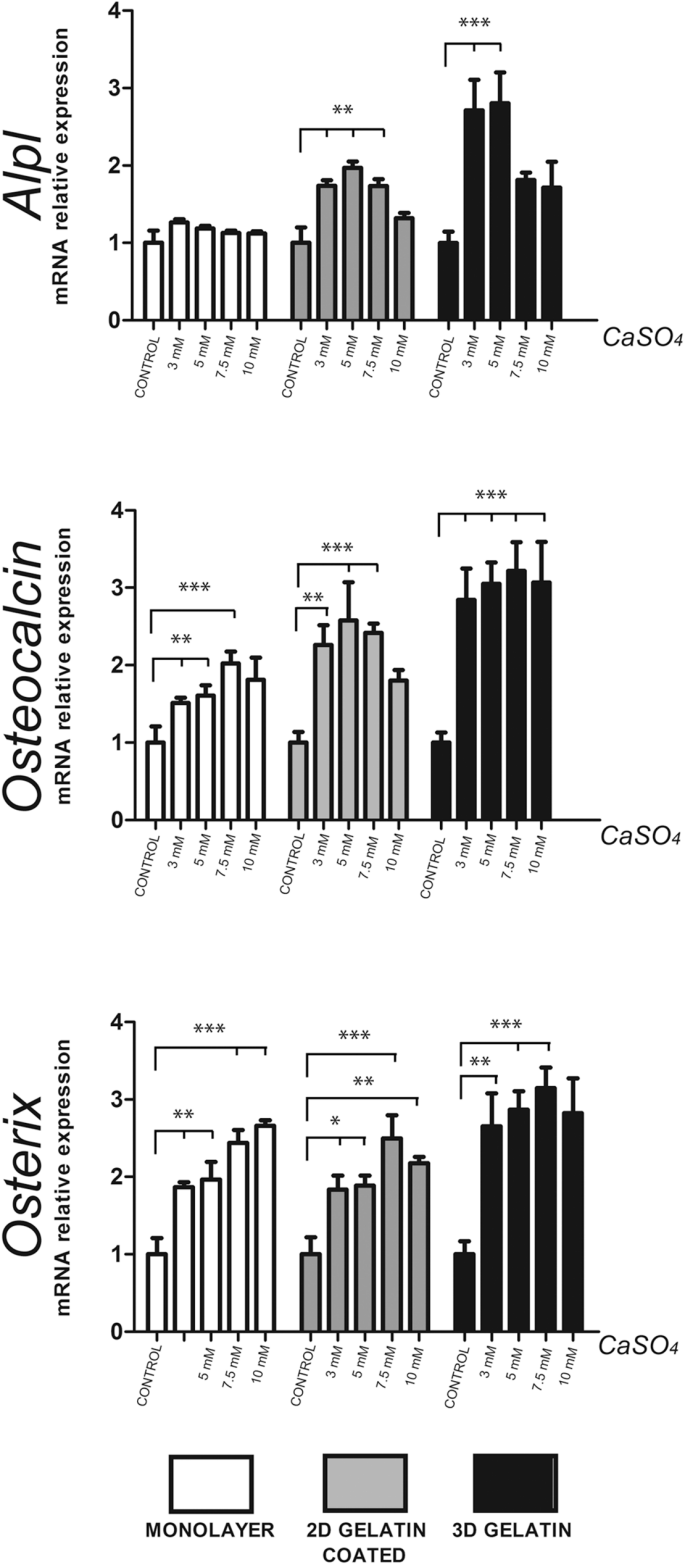
Extracellular calcium induces expression of osteogenic markers on bone marrow mesenchymal stem cells. Three different culture models were compared (cells in monolayer in plastic surface, cells in monolayer in gelatin-coated dishes and cells in 3D gelatin scaffolds). After 10 days, the mRNA expression of *Alpl*, *Osteocalcin* (*Bglap2*) and *Osterix* was analysed and normalised to *Gapdh* levels (n = 3). Differences were considered significant at p values: * p < 0.05,** p < 0.01, and ***p < 0.001.

We then assessed whether extracellular Ca^2+^ could have a beneficial effect on osteoblast differentiation. We evaluated the effects of different Ca^2+^ concentrations on BM-MSCs using the three culture systems described above. Higher expressions of *Alpl*, *Osteocalcin* and *Osterix* were obtained using Ca^2+^ concentrations from 3 mM to 10 mM (Fig 1). A concentration of 7.5 mM was optimal for the late osteogenic differentiation markers *Osteocalcin* and *Osterix*. Taken together, these results suggest that extracellular Ca^2+^ concentrations of between 3 mM and 10 mM produce a beneficial effect on the expression of *Osteocalcin* and *Osterix*, regardless of the culture model used. We confirmed the specificity of these effects by comparing CaSO_4_ and CaCl_2_ as calcium ion sources or by chelating Ca^2+^ with EDTA. Both CaSO_4_ and CaCl_2_ stimulated the expression of osteogenic markers. Moreover, the addition of EDTA completely blocked the positive effects of CaSO_4_ and CaCl_2_ on gene expression (S2 Fig).

### Cooperation of calcium and BMP-2 in osteoblast differentiation and bone regeneration of calvarial critical-size defects in mice

**Fig 2:**
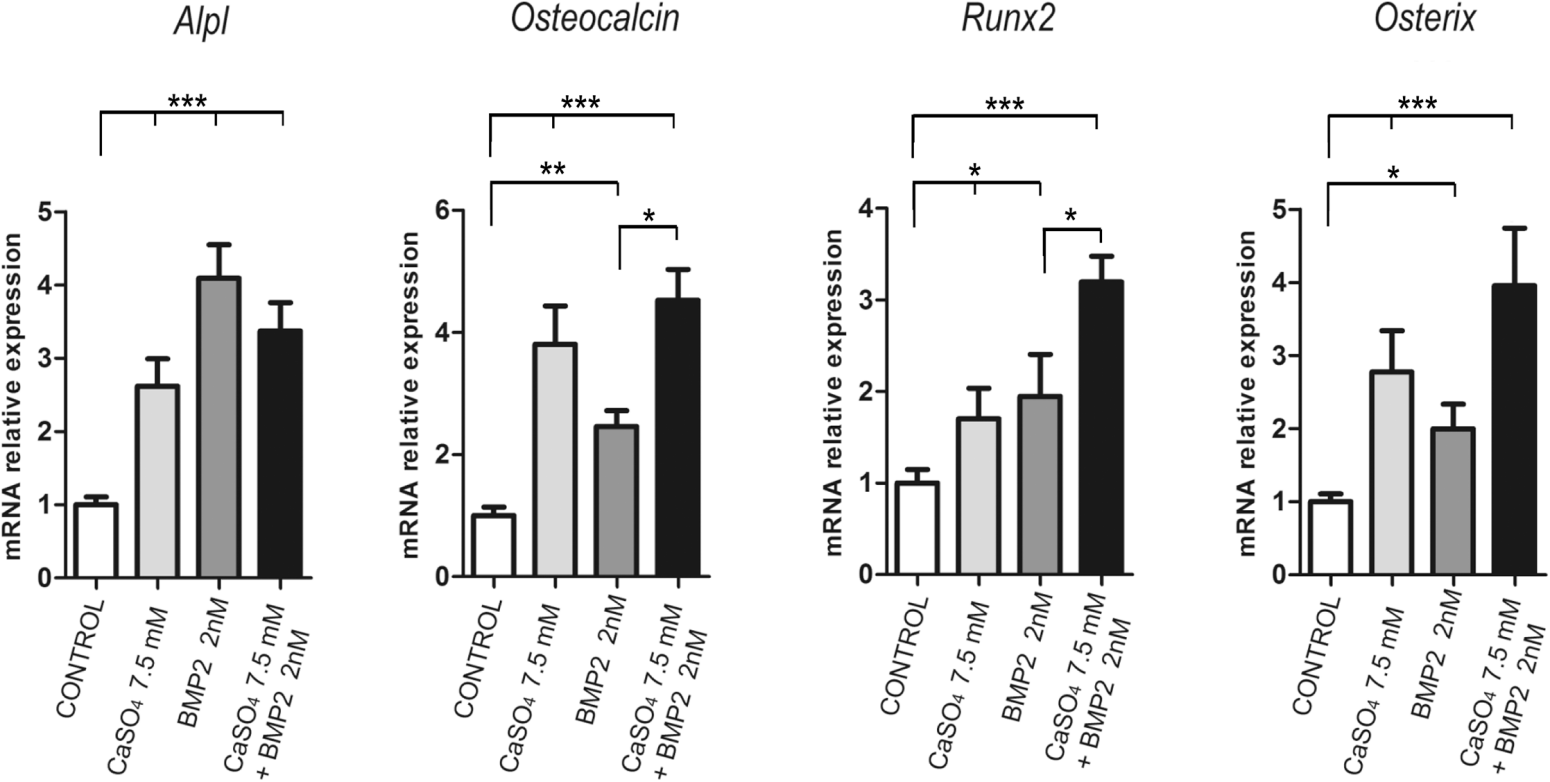
Extracellular calcium increases the effects of BMP-2 on osteogenic marker expression. Primary BM-MSCs were cultured on 3D gelatin scaffolds with 7.5 mM of CaSO_4_ and/or 2 nM of BMP-2 for 10 days. The mRNA expression of *Alpl*, *Osteocalcin* (*Bglap2*), *Runx2* and *Osterix* was analysed and normalised to *Gapdh* levels (n = 3). Differences were considered significant at p values: * p < 0.05,** p < 0.01, and ***p < 0.001.

We further evaluated whether Ca^2+^ would cooperate with osteoinductive cytokines such as BMPs. BM-MSCs were cultured in a 3D gelatin scaffold and stimulated with 7.5 mM CaSO_4_ and/or BMP-2 (2 nM). Incubation for 10 days with CaSO_4_ or BMP-2 alone promoted expression of all the bone markers analysed (Fig 2). More importantly, although a combination of Ca^2+^ and BMP-2 did not present any additive effects on the expression of the early osteogenic marker *Alpl*, they produced a significant additive effect on the expression of *Osteocalcin*, *Runx2* and *Osterix* (Fig 2). Thus, when added to culture media, Ca^2+^ exerts a cooperative action with BMP-2 on late osteogenic marker expression.

**Fig 3:**
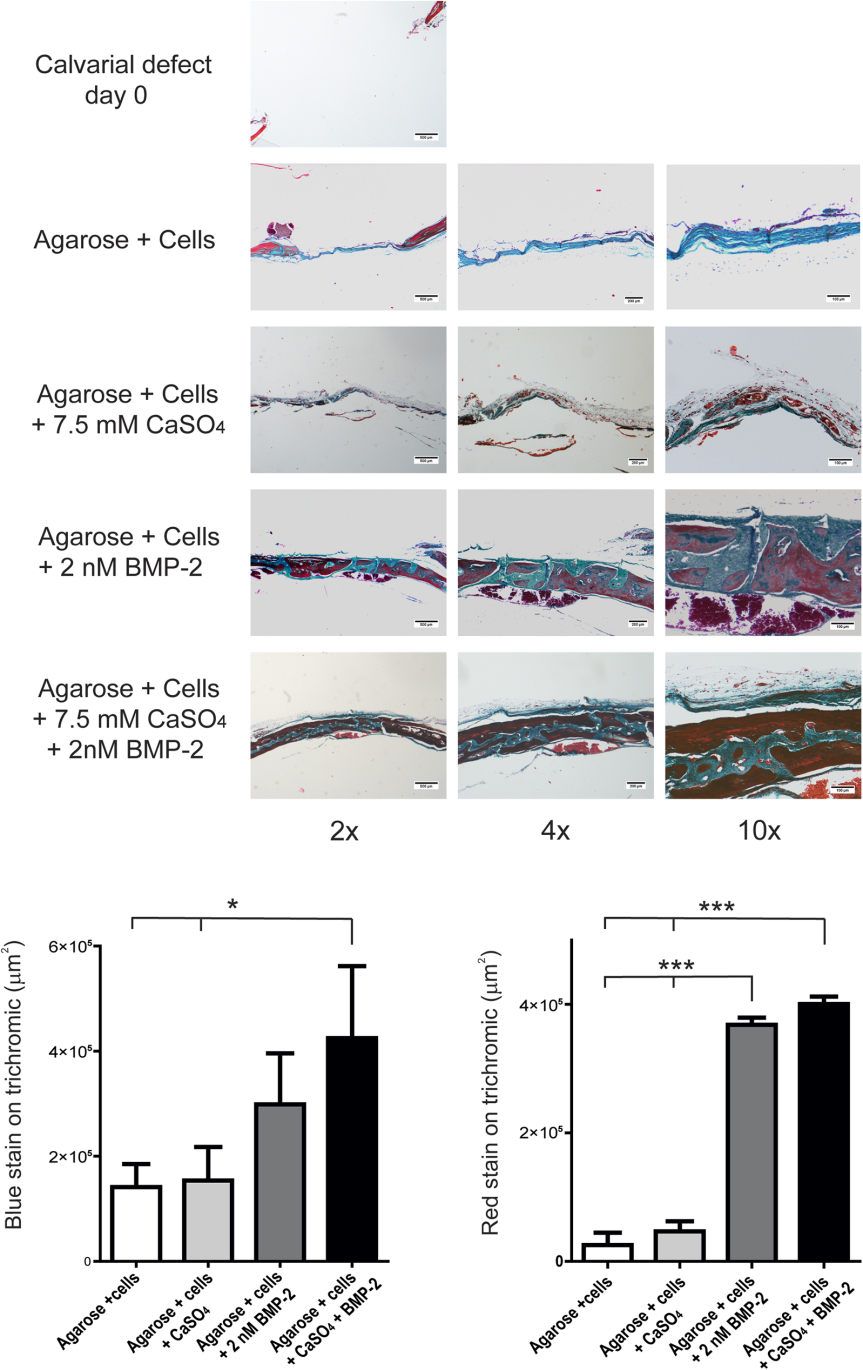
Extracellular calcium increases the effects of BMP-2 on bone regeneration *in vivo*. Calvarial critical-size bone defects were generated in mice and implanted with cells cultured in 3D scaffolds pre-treated with 7.5 mM of CaSO_4_ and/or 2 nM of BMP-2 for 48 hours. After five weeks, the implanted constructs were retrieved and processed for Masson’s trichrome staining. Scale bars are shown for the different magnifications. Lower panels show the relative quantification of the areas of new bone formation from the 4x magnification stained by thrichrome in red or blue (n = 3).

To extend our *in vitro* results on the cooperation between Ca^2+^ and BMPs to an *in vivo* context, we analysed bone formation in calvarial critical-size bone defects in mice. Five-millimetre defects were performed in parietal bones and further implanted with BM-MSCs previously seeded in 3D gelatin scaffolds and pre-treated for 48 hours with either 7.5 mM CaSO_4_ or 2 nM BMP-2 alone or combined. After five weeks, skulls were retrieved and analysed for bone formation in the defect. Masson’s trichrome stains showed dense connective tissue but no major bone formation in the control group. Higher levels of mineralisation and bone maturation were found in those implants treated with BMP-2. Similarly, higher bone formation took place when implants were pre-treated by combination of CaSO_4_ and BMP-2 (Fig 3). Moreover, a combination of CaSO_4_ and BMP-2 led to a more mature bone structure. Both osteoblast and osteocytes can be observed in these bone regeneration areas (Fig 3).

### Signalling pathways involved in the cooperation of calcium and BMP-2 during osteogenesis of BM-MSCs

**Fig 4:**
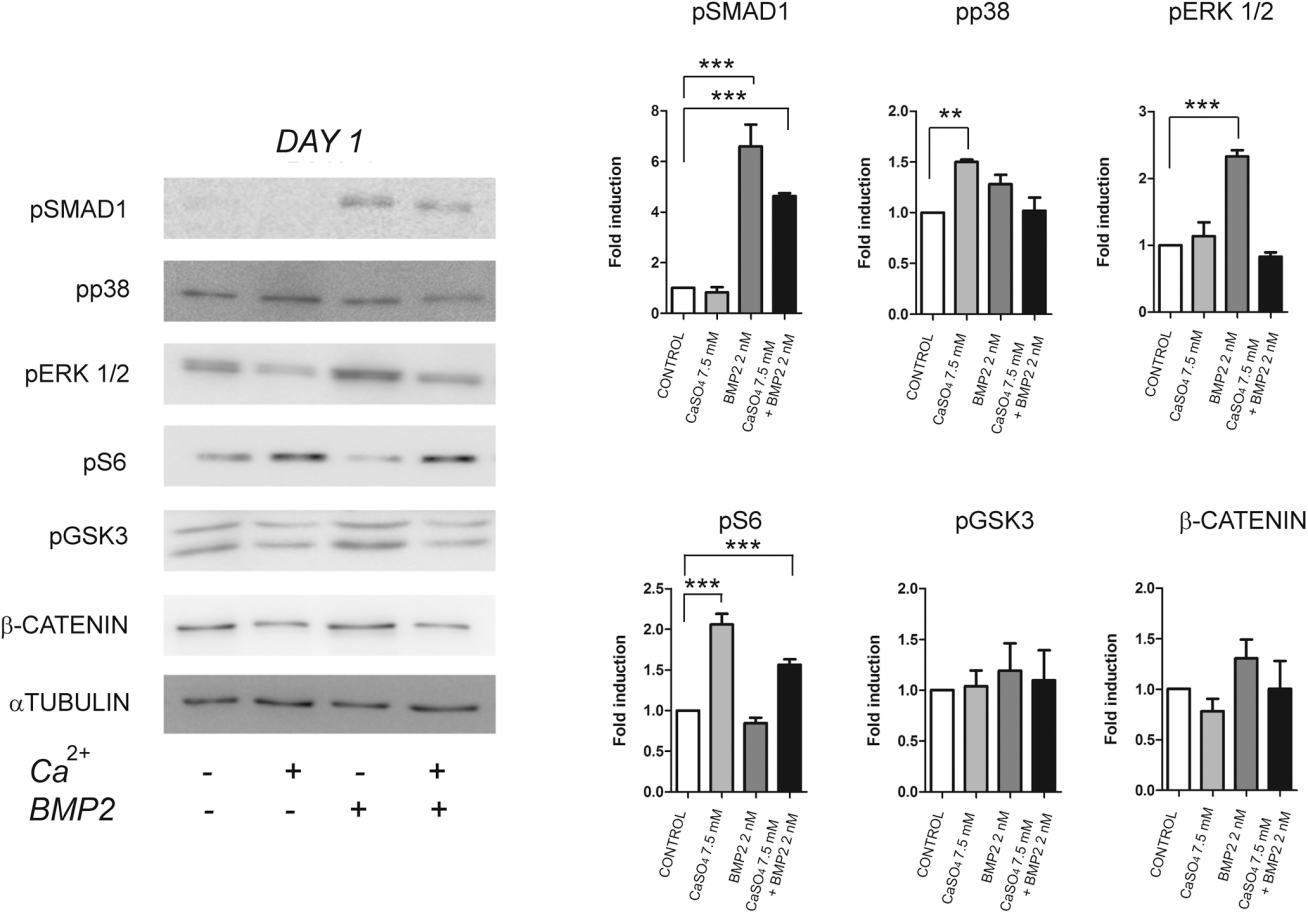
Early effects of extracellular calcium on cell signalling. Cells were cultured in 3D gelatin scaffolds with Ca^2+^ (7.5 mM) and/or BMP-2 (2nM) for 24 hours and extracts analysed by Western blot. Data was quantified relative to the levels of α-TUBULIN (n = 3). Differences were considered significant at p values: * p < 0.05,** p < 0.01, and ***p < 0.001.

To determine the mechanisms of cooperation between extracellular calcium and BMP-2 in BM-MSCs differentiation, we analysed intracellular signalling triggered by both signalling molecules at early and late differentiation points. Early analysis was performed 24 hours after CaSO_4_ and/or BMP-2 stimulation. As expected, BMP-2 promoted phosphorylation of SMAD1/5 and increased the levels of phosphorylated ERK1/2 (Fig 4). By contrast, p38 and S6-kinase (S6K) signalling pathways were activated when cells were treated with Ca^2+^ alone. It is worth noting that, at this initial differentiation stage, an antagonistic effect on each signalling pathways was obtained when Ca^2+^ was added together with BMP-2 (Fig 4).

**Fig 5:**
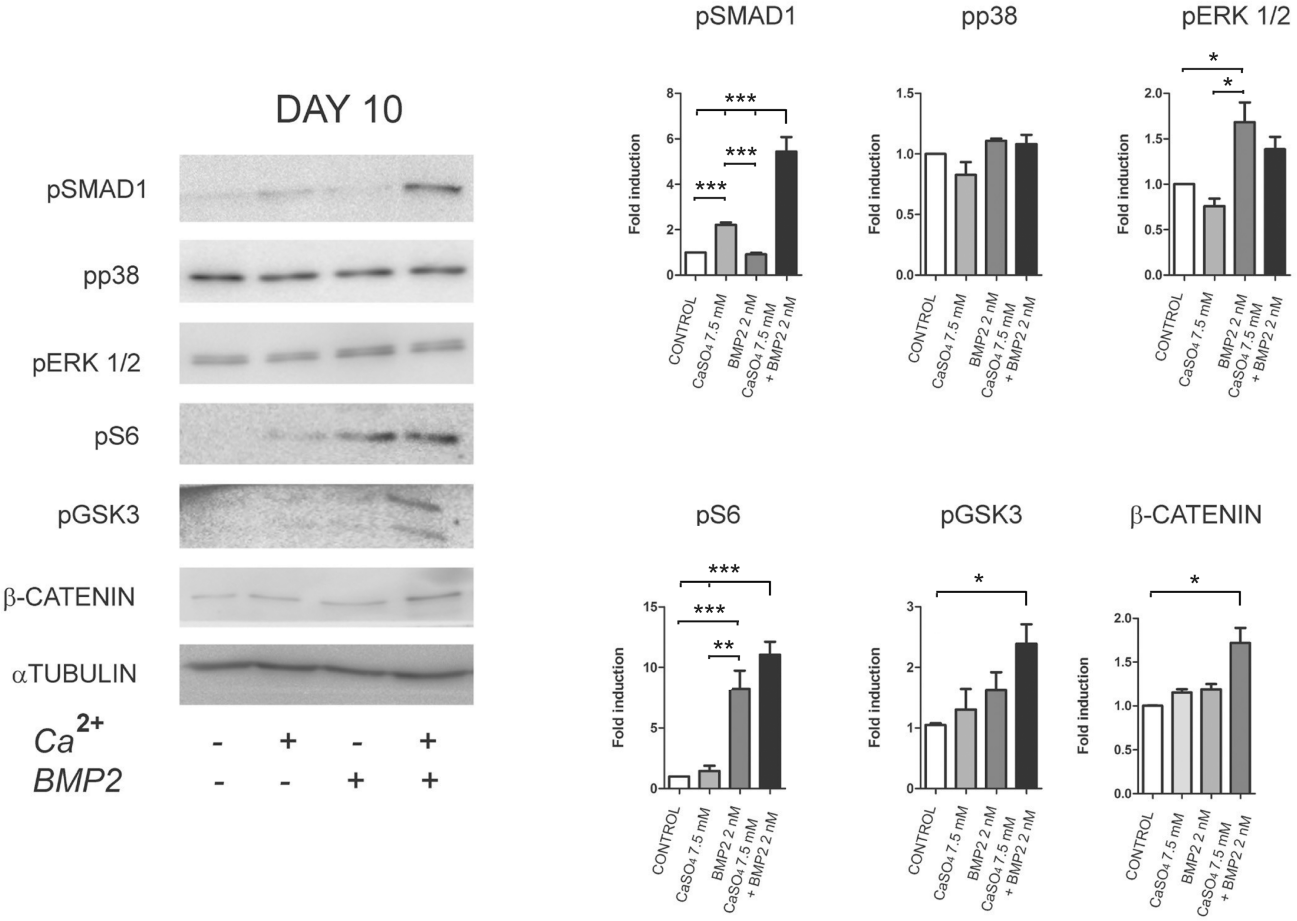
Late effects of extracellular calcium on cell signalling. Cells were cultured in 3D gelatin scaffolds with Ca^2+^ (7.5 mM) and/or BMP-2 (2nM) for 10 days. Data was quantified relative to the levels of α-TUBULIN (n = 3). Differences were considered significant at p values: * p < 0.05, ** p < 0.01, and ***p < 0.001.

The same intracellular components were subsequently assessed after treatment with a combination of CaSO_4_ and/or BMP-2 for 10 days. A significant additive or cooperative effect between Ca^2+^ and BMP-2 was observed on the phosphorylation levels of SMAD1/5 (Ser463-465), S6 (Ser235-236), GSK3β (Ser9) and the total levels of β-CATENIN (Fig 5). Phosphorylation at Ser9 of GSK3β is mediated by AKT and results in the inhibition of its β-CATENIN repression action. Thus, these results suggest that extracellular calcium produces a differential time-dependent effect on BMP-2 and AKT signalling. A signalling network antagonistic to BMP-2 is activated early on, whereas Ca^2+^ promotes a cooperative effect on several intracellular signalling events later on.

### Extracellular calcium promotes endogenous secreted BMP-2 and BMP-4 mRNA expression

**Fig 6:**
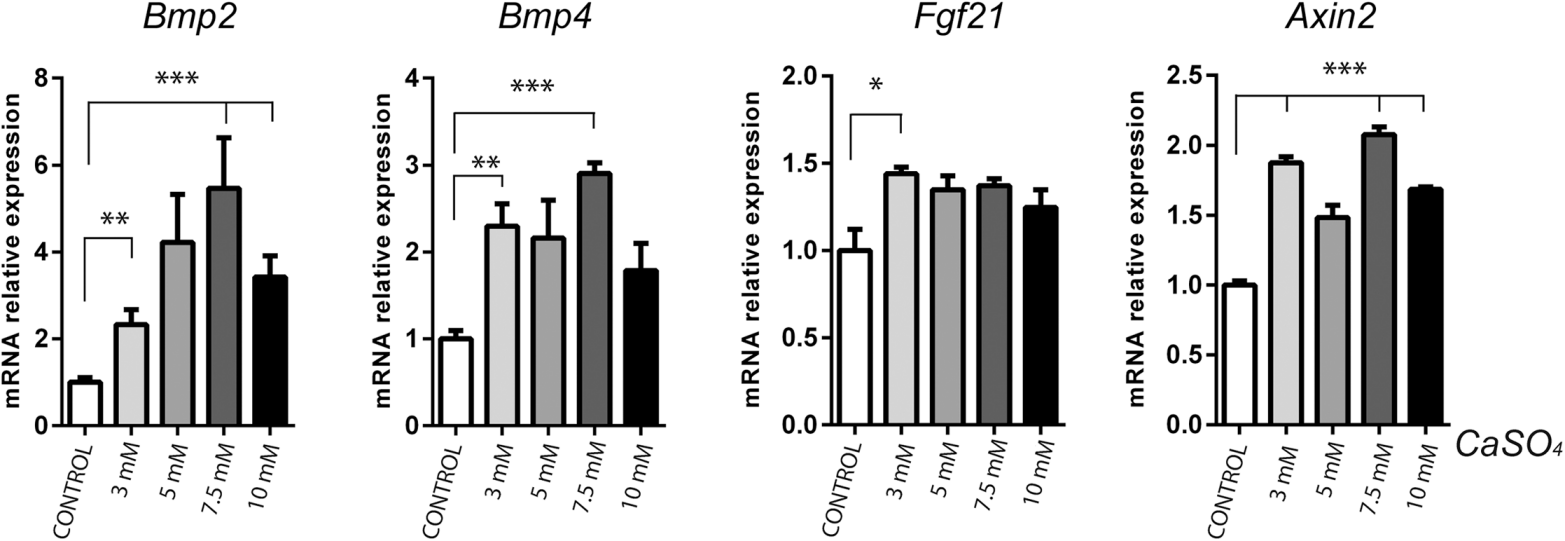
Extracellular calcium induces *Bmp2*, *Bmp4*, *Fgf21* and *Axin2* mRNA expression. BM-MSCs were cultured on 3D gelatin scaffolds with different extracellular calcium concentrations for 10 days. The mRNA expression of *Bmp2*, *Bmp4*, *Fgf21* and *Axin2* was analysed and normalised to the levels of *Gapdh* (n = 3). Differences were considered significant at p values: * p < 0.05,** p < 0.01, and ***p < 0.001.

After 10 days of differentiation, BM-MSCs stimulated with calcium alone showed activation of the SMAD1/5 pathway (Fig 5). Since there is no evidence that calcium activates BMP receptors directly, it could be suggested that the increased availability of BMP receptor ligands was responsible. We therefore hypothesised that, once these cells are committed to the osteoblast lineage, Ca^2+^ induces cells to secrete endogenous factors that reinforce differentiation through an autocrine/paracrine mechanism. We assayed whether Ca^2+^ induced BMP-2 or BMP-4 expression. BM-MSCs cultured in 3D gelatin scaffolds were exposed to CaSO_4_ concentrations (from 3 mM to 10 mM) for 10 days. An increase, which reached its maximum at 7.5 mM, was obtained for both *Bmp2* and *Bmp4* mRNA expression (Fig 6). In addition, we also determined the mRNA levels of *Fgf21* (a tyrosine kinase receptor ligand that inhibits osteoblastogenesis[22] and *Axin2* (a target of the Wnt/β-CATENIN pathway downstream of GSK3). A significant increase in *Axin2* expression was found, in line with *Bmp2* and *Bmp4* mRNA expression. By contrast, *Fgf21* mRNA expression was only slightly elevated, without any dose-response effect (Fig 6). Taken together, these results demonstrate that BM-MSCs stimulated with Ca^2+^ secrete higher levels of multiple critical cytokines that amplify their osteoblastic differentiation response.

## Discussion

Different anatomical zones are used to obtain autogenous bone grafts to reconstruct bone defects. These sources include the iliac crest, cranial bone, mandibular symphysis, rib and tibia [23]. However, drawbacks include limited availability and morbidity at the donor site. To overcome these disadvantages, numerous tissue engineering approaches have been developed to take advantage of physiological osteoinductive signals [24]. Both Ca^2+^ and BMP-2 are known to be co-released into the extracellular space by osteoclasts after bone matrix resorption. Our hypothesis was that extracellular Ca^2+^ signals interact with BMP and lead to higher osteoblast differentiation and bone formation from BM-MSCs. Here, early and late osteogenic marker expression and histological assessment of bone regeneration in a calvarial critical-size defect model demonstrated that extracellular Ca^2+^ enhances the effects of BMP-2 on *Osteocalcin*, *Runx2* and *Osterix* expression and promotes bone regeneration *in vivo*. More importantly, mechanistically, both osteoinductors combined cooperate to increase long-term activation of SMAD and AKT signalling.

Osteogenic gene expression was significantly higher when extracellular Ca^2+^ was added, regardless of the cell culture system used. MG63 osteoblastic cells, both in monolayer culture and 3D gelatin hydrogels, have been reported to show significant mineralisation when cultured with 8 mM Ca^2+^ [25]. Moreover, BM-MSCs treated simultaneously with two different sources of Ca^2+^, CaSO_4_ and CaCl_2_, with or without EDTA, demonstrated that the osteogenic effect was specific for Ca^2+^. This outcome is consistent with the EGTA inhibition of osteocalcin secretion [26] and BAPTA, an intracellular Ca^2+^ chelator, in the response of osteoblasts to extracellular calcium [27].

Osteoinductive factors released from resident cells or after osteoclast bone resorption regulate the recruitment and differentiation of osteoblastic progenitor cells. The binding of BMPs to their cognate receptors triggers canonical Smad and Smad-independent pathways, including ERK, p38 and PI3K/AKT signalling [11,28]. Several authors have reported that CaSR activation by extracellular Ca^2+^ also activates these same pathways [4,27,29]. We found that both calcium and BMP-2 induce activation of common signalling components, but in a differential time-dependent response. An early antagonistic effect between Ca^2+^ and BMP-2 signalling was demonstrated. This contrasting effect is consistent with previous reports showing a crosstalk between calcium signalling and BMP pathway in which high intracellular calcium inhibits BMP signalling [30]. Furthermore, Ca^2+^/calmodulin dependent kinase II (CAMKII), a primary transducer of calcium ions, directly interacts with SMADs and antagonises their function [31]. This reverse effect could be reinforced when we also consider that MAPK signalling inhibits BMP signals at the level of SMAD1 [32]. Osteoblast differentiation is a multistep cascade of gene expression that initially supports proliferation and survival [33]. The early prominent proliferative and pro-survival role of the MAPK and AKT/S6K network could induce the enlargement of the osteogenic progenitor pool[4].

However, when late differentiation events were analysed, Ca^2+^ and BMP-2 were found to cooperatively stimulate osteoblast differentiation through the strengthening of specific osteogenic signalling pathways. Increases in the phosphorylation of SMAD1/5, S6, GSK3β and expression of β-CATENIN were consistent with the significantly higher expression of *Osteocalcin*, *Runx2* and *Osterix* and the greater bone formation *in vivo* [34,35]. Cooperative crosstalk between Ca^2+^ and BMP-2 in osteoblasts through the induction of the calcium-dependent transcription factor NFATc1 by BMP-2 has also been described recently [36]. NFAT transcription factors have proved necessary for osteoblast differentiation and bone formation [37]. Mechanistically, NFAT transcription factors activate osteogenesis through their interaction with OSX and their ability to stimulate Wnt/β-CATENIN signalling [37,38]. Unexpectedly, cell cultures exposed to Ca^2+^ alone for 10 days displayed significantly higher SMAD signalling. This observation correlated with the increase in *Bmp2*, *Bmp4* and *Axin2* gene expression. BM-MSCs, periodontal ligament cells and dental pulp cells exposed to calcium-derived biomaterials have been reported to induce the upregulation of *Bmp2* mRNA expression [8,39,40]. These studies implicated MAPK activity and AP-1 transcription factors in such effects [8,41]. Thus, our data support the suggestion that calcium induces an autocrine/paracrine loop by endogenous BMP upregulation. Furthermore, *Axin2* (a target gene of Wnt/β-CATENIN signalling downstream of GSK3β) was also expressed in long-term cell cultures. Our group recently showed that PI3K/AKT activity is relevant in bone formation *in vivo* and leads to the activation of SMAD1/5 and GSK3β/β-CATENIN signalling [35]. Several reports have demonstrated the synergistic interaction and the significance between BMP and Wnt during osteoblast differentiation and bone formation *in vitro* and *in viv*o [21,34]. Taken together, our results demonstrate a delayed calcium signalling effect that likely integrates and reinforces an osteogenic programme from multiple inputs.

## Supporting information

S1 Fig 3D gelatin scaffolds positively influence the expression of osteogenic markers. Three different culture models were compared (cells cultured in monolayer in plastic surface, cells cultured in monolayer in gelatin-coated dishes and cells cultured in 3D gelatin scaffolds). Primary BM-MSCs were cultured in each system for 10 days. The mRNA expression of *Alpl*, *Osteocalcin* (*Bglap2*) and *Osterix* was analysed and normalised to the levels of *Gapdh* (n = 3).
(PDF)

S2 Fig EDTA abrogates the effects of calcium on osteogenic marker expression. BM-MSCs cultured in 3D gelatin scaffolds were exposed to CaSO_4_ and CaCl_2_ as a source of extracellular calcium (7.5 mM) with or without EDTA (7.5 mM). After 10 days, the mRNA expression of *Osteocalcin* (*Bglap2*), *Runx2* and *Osterix* was analysed and normalised to the levels of *Gapdh* (n = 3).
(PDF)
