# Supplementary figures and images for: Extracellular calcium promotes bone formation from bone marrow mesenchymal stem cells by amplifying the effects of BMP-2 on SMAD signalling
Source: PLoS One. 2017 May 25;12(5):e0178158. doi: 10.1371/journal.pone.0178158 (PMC5444778; doi:10.1371/journal.pone.0178158)

*Alpl*

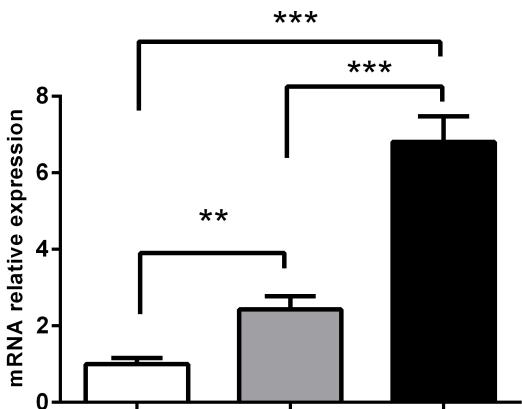

*Osteocalcin*

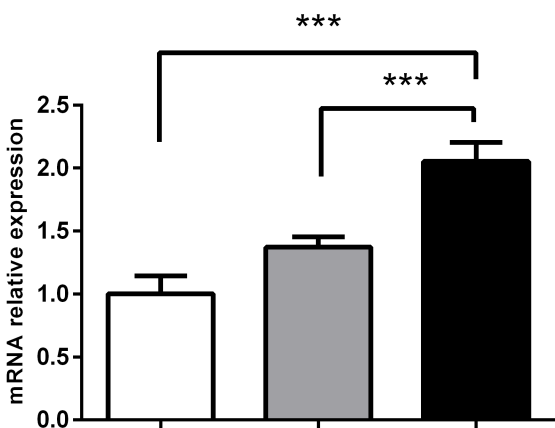

*Osterix*

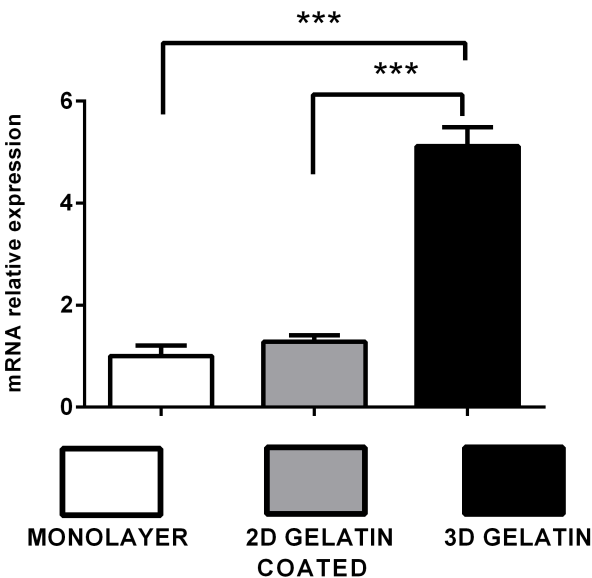

Supplement: S1 Fig — Three different culture models were compared (cells cultured in monolayer in plastic surface, cells cultured in monolayer in gelatin-coated dishes and cells cultured in 3D gelatin scaffolds). Primary BM-MSCs were cultured in each system for 10 days. The mRNA expression of Alpl, Osteocalcin (Bglap2) and Osterix was analysed and normalised to the levels of Gapdh (n = 3). (PDF) [file pone.0178158.s001.pdf]

## *Osteocalcin*

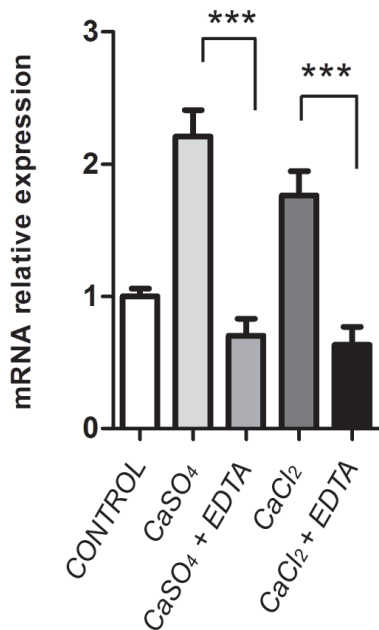

## *Runx2*

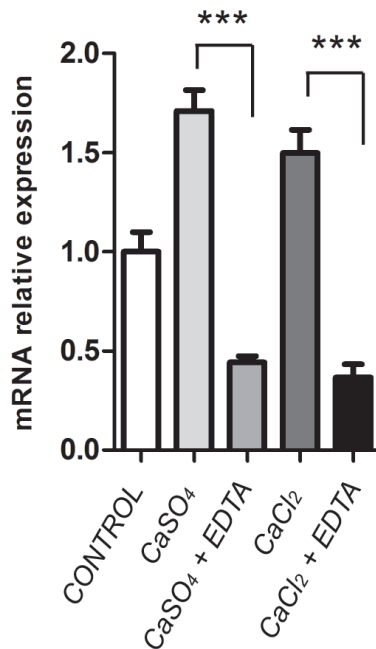

## *Osterix*

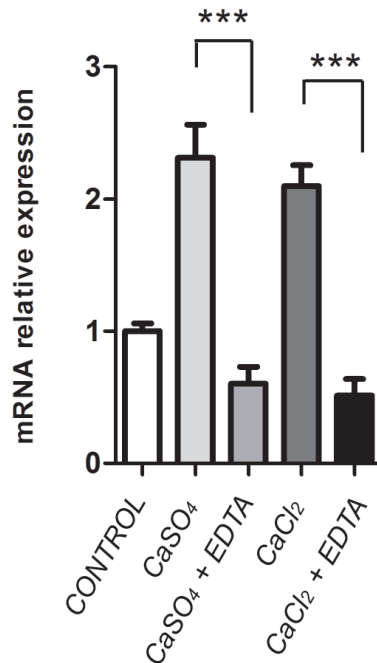

Supplement: S2 Fig — BM-MSCs cultured in 3D gelatin scaffolds were exposed to CaSO4 and CaCl2 as a source of extracellular calcium (7.5 mM) with or without EDTA (7.5 mM). After 10 days, the mRNA expression of Osteocalcin (Bglap2), Runx2 and Osterix was analysed and normalised to the levels of Gapdh (n = 3). (PDF) [file pone.0178158.s002.pdf]
